# A Customizable Platform to Integrate CAR and Conditional Expression of Immunotherapeutics in T Cells

**DOI:** 10.3390/ijms251910568

**Published:** 2024-09-30

**Authors:** Huong T. X. Nguyen, Yabin Song, Satendra Kumar, Fu-Sen Liang

**Affiliations:** 1 Department of Chemistry, Case Western Reserve University, 2080 Adelbert Road, Cleveland, OH 44106, USA; hxn119@case.edu (H.T.X.N.); sxk2095@case.edu (S.K.); 2 Department of Chemistry and Chemical Biology, University of New Mexico, Albuquerque, NM 87106, USA; nksong@gmail.com

**Keywords:** cancer immunotherapy, tumor microenvironment, cancer signal, solid tumors, small molecule, CIP, immunomodulators, CAR-T cell

## Abstract

The potential of chimeric antigen receptor (CAR)-based immunotherapy as a promising therapeutic approach is often hindered by the presence of highly immunosuppressive tumor microenvironments (TME). Combination therapies with either co-administration or built-in expression of additional TME-modulating therapeutic molecules to potentiate the functions of CAR-T cells can cause systemic toxicities due to the lack of control over the delivery of biologics. Here, we present a proof-of-concept engineered platform in human Jurkat T cells that combines CAR with a therapeutic gene circuit capable of sensing *β*-galactosidase (a reported cancer-associated signal) and subsequently activate the production of customized therapeutic gene products. We have demonstrated the integration of the chemically induced proximity (CIP) and associated signal sensing technologies with CAR in this study. A *β*-galactosidase-activatable prodrug was designed by conjugating a galactose moiety with a CIP inducer abscisic acid (ABA). We showed that Jurkat T cells engineered with CAR and the ABA-inducible genetic circuits can respond to recombinant *β*-galactosidase to drive the production and secretion of various immunotherapeutics including an anti-cancer agent, an immunomodulatory cytokine, and immune checkpoint inhibitors. Our design is highly modular and could be adapted to sense different cancer-related signals to locally produce antitumor therapeutics that can potentially boost CAR-T efficacy and persistence.

## 1. Introduction

The potential of cancer immunotherapy using engineered T cells with chimeric antigen receptors (CAR) to eradicate targeted tumors has been demonstrated in several clinical trials and FDA approvals. Although CAR-redirected T-cell therapies have been successful in treating hematological malignancies, their application against solid tumors has proven challenging. One hindering factor is the difficulty of identifying tumor-specific antigens [1]. Current cancer-associated antigens are not exclusively expressed on tumor cells. When expressed in normal tissue, even a low-level expression could mount a devastating effect [2]. Even if a unique antigen for a solid tumor could be identified, T cells unfortunately become exhausted within the repressive tumor microenvironment (TME) [3]. The TME of solid tumors suppresses the proliferation of engineered T cells and their production of cytokines in favor of promoting tumor development. To overcome this hurdle, many strategies have been developed to potentiate the therapeutic functions of CAR-T cells against solid tumors. One approach involves the co-administration of CAR-T cells with additional therapeutic molecules capable of modulating the TME and CAR-T cell activity, such as cytokines or immune checkpoint inhibitors. However, such combination therapies can cause systemic toxicities due to the lack of control over the distribution of therapeutic effects [4]. Alternatively, many efforts have been dedicated to engineering “armored” CAR-T cells capable of the local delivery of biologics to alleviate systemic side effects. This has led to the development of T-cell redirected universal cytokine killing (TRUCK), an engineered CAR with tethered nuclear factor of activated T-cell (NFAT)-induced cytokine expression and secretion [5]. With TRUCKs, the CAR engagement of a cognate antigen leads to the activation of CAR and NFAT signaling, resulting in the NFAT-driven expression and subsequent secretion of cytokines, while without CAR activation, no cytokine release can occur. These cytokine-producing CAR-T cells have shown enhanced efficacy when delivering IL-12 [6,7], IL-15 [8], IL-18 [9], or IL-21 [10] to the TME. Recently developed “synNotch” T cells demonstrated a general platform to conditionally deliver custom response programs to remodel the local microenvironment, in response to a user-selected antigen [11]. Although these designs can provide a flexible way to engineer a customized and localized immune response program, such systems are greatly dependent on antigen-driven T-cell activation, which may not be effective when tumors lose or downregulate their antigen expression as a mechanism of tumor immune escape. Also, the inevitable antigen expression in normal tissues can result in off-tumor toxicity mediated by the non-selective delivery of biologics and enhancement of CAR-T cell activities.

**Figure 1:**
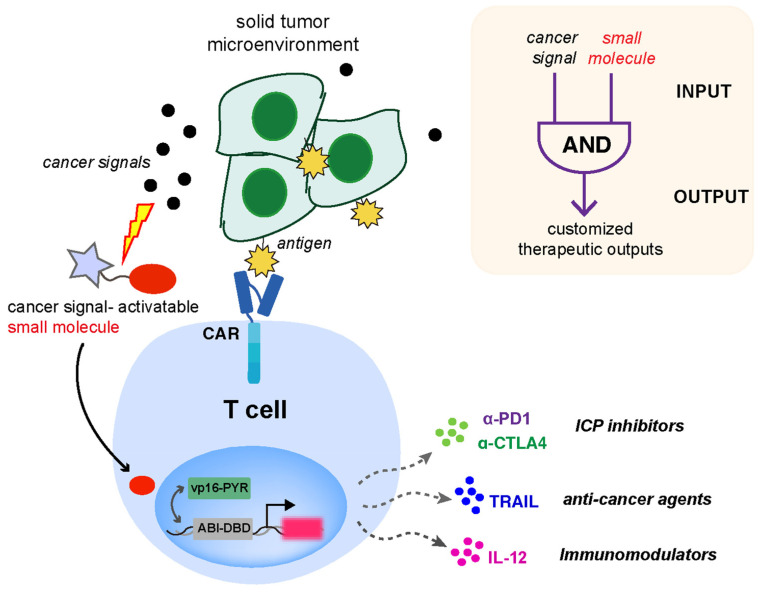
The design of CAR and inducible gene circuit integrated T cell platform. The programmable in situ production of therapeutic outputs is designed to be triggered by cancer-associated signals to enhance the efficacy and persistence of CAR-expressing T cells.

To overcome the suppressive TME and address off-tumor toxicity problems encountered in current CAR-T therapies, we present a new strategy in this paper that we envision can be applied to empower CAR-T cells to modulate the immune response program, independent of the antigen recognition by CAR. We expect that discriminating cancer cells based on the incorporation of additional cancer markers (e.g., TME signals) will improve the specificity of antitumor biologics delivery. To demonstrate the feasibility of this strategy, we coupled CAR with a parallel customized T-cell response program built upon an “AND” logic gate design using integrated small molecule-induced protein dimerization and inducer caging strategies (to allow TME signal sensing and generating therapeutic responses). A previously developed small molecule-induced gene expression system based on the abscisic acid (ABA)-based chemically induced proximity (CIP) technology provides an inducible and titratable way to control various biological behaviors in cells [12]. CIP methods use chemical inducers (e.g., ABA) to selectively trigger the heterodimerization of two adaptor proteins (e.g., ABI and PYL/PYR for ABA) that can be genetically fused to two proteins of interest to control downstream cellular effects (e.g., gene expression). Moreover, the ABA caging strategy that involves the chemical modification of ABA with different sensing units activatable by custom signals (e.g., H_2_O_2_, Fe^2+^, or light) to drive different cellular responses has been successfully implemented [13,14,15,16]. We hypothesized that this platform can be incorporated into immune cells engineered with a CAR to gain enhanced therapeutic efficacy through tailored cancer-inducible immunomodulatory gene circuits. This strategy will contribute to the generation of “armored” CAR-T cells capable of the local delivery of biologics to overcome immunosuppressive TME, which, however, are not dependent on CAR activation but on specific TME signals. To test this strategy, an ABA-responsive split transcriptional and an inducible gene cassette expressing secreted therapeutics were incorporated into CAR-expressing human Jurkat T cells. The chosen cancer-associated signals are expected to activate caged ABA and consequently induce gene expression through the ABA-triggered dimerization and activation of the split transcriptional activator (Figure 1). In the setting of CAR-T-based immunotherapies, achieving the localized release of custom biologics is expected to reduce systemic toxicities and improve the therapeutic efficacy of the reprogrammed T cells within TME.

The TME of solid tumors is known to have abnormal levels of various biological signals such as overexpressed enzymes, reduced pH, and oxygenation. Developing prodrugs responding to these TME-specific signals is a common strategy by caging cytotoxic reagents with moieties that inactivate the drugs but can be removed by tumor-specific signals, which lead to the local release of active therapeutic molecules [17]. *β*-galactosidase (*β*-gal) is one of the common lysosomal glucosidases, which hydrolyzes the terminal galactose in glycoconjugates and has been reported to be an important biomarker overexpressed in senescent cells and ovarian cancer cells [18,19]. As a proof-of-concept study to apply the signal-triggered CIP strategy in the cancer context, we developed an ABA prodrug bearing a galactose moiety that can be activated by *β*-gal. We demonstrated that this signal-induced CIP technology can be used to engineer Jurkat T cells capable of sensing cancer-related signals (e.g., *β*-gal) to selectively deliver desired therapeutic payloads. The outputs can be any genetically encodable therapeutics (including anti-cancer agents, secreted stimulatory cytokines, and immune checkpoint inhibitors) that are expected to boost the antitumor activity of CAR-T cells and improve CAR-T cells’ persistence within the tumor milieu. We believe that the unparalleled programmability of this conditional synthetic gene regulatory circuit can be broadly applied against different cancers whose microenvironment-associated signals are exclusively elevated.

## 2. Results and Discussion

### 2.1. Engineering ABA-Inducible Therapeutics Expression System in HEK-293T Cells

**Figure 2:**
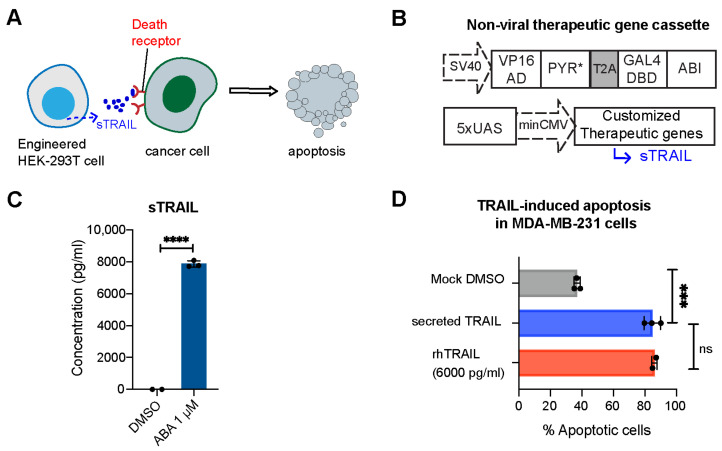
ABA-induced production of sTRAIL in engineered HEK-293T cells. (**A**) Cartoon illustrates sTRAIL-induced apoptosis in death receptor-expressing cancer cells. (**B**) ABA-inducible therapeutic gene expression constructs. (**C**) Induction of sTRAIL in HEK-293T cells in response to ABA (1 μM). The concentration of secreted TRAIL in the cell culture supernatant was measured by ELISA. (**D**) Percentage of MDA-MB-231 cell apoptosis after being treated with DMSO, recombinant human TRAIL (rhTRAIL), or media containing sTRAIL for 24 h. Target cells were co-stained with Annexin V and propidium iodide to exclude necrotic dead cells. Data are calculated from 3 biological replicates and presented as mean ± S.E.M. **** *p* ≤ 0.0001, *** *p* ≤ 0.001, ns, *p* ≥ 0.05, two-tailed Student *t*-test.

To develop T cells capable of locally delivering desired therapeutic effects, we first constructed an ABA-inducible gene expression system controlling the expression of a therapeutic gene encoding secreted tumor necrosis factor-related (TNF) apoptosis-inducing ligand (sTRAIL) (Figure 2A,B). TRAIL is a selective inducer of apoptosis in many transformed cells bearing death receptors and has been a promising candidate for cancer therapy [20,21,22]. This ABA-inducible gene expression system uses ABA to induce the heterodimerization of the ABI-fused yeast GAL4 DNA-binding domain (GAL4DBD) to the PYL/PYR-fused herpes simplex virus VP16 transactivation domain (VP16AD). The ABA-induced reconstitution of functional transcriptional activator will lead to the expression of GAL4DBD-targeted genes (e.g., sTRAIL) [12]. The PYR mutant (PYR*) developed in our previous research [16] was incorporated to offer an enhanced ABA potency. We co-transfected HEK-293T cells with the plasmids of ABA-inducible sTRAIL and the split transcriptional activator (VP16AD-PYR*-T2A-GAL4DBD-ABI) for 24 h, and then treated cells with or without ABA. At 24 h post-treatment, the secretion of sTRAIL in culture media was quantified by the enzyme-linked immunosorbent assay (ELISA). Cells that are treated with ABA secreted a high level of TRAIL, as shown in Figure 2C. To validate the biological activity of secreted TRAIL, MDA-MD-231 cells (expressing the death receptor) were incubated with culture media containing ABA-induced sTRAIL or commercial recombinant human TRAIL for 24 h, and the death of target cells (as a result of apoptosis) was measured by flow cytometry via annexin V-FITC staining. Propidium iodide (PI) staining was used to quantify necrotic cells and differentiate from the apoptotic cells. We observed an increase in apoptotic cells when cancer cells were incubated with ABA-induced sTRAIL, which showed comparable efficacy as the case when treated with commercial recombinant human TRAIL (Figure 2D). These results confirmed the feasibility of this ABA-inducible expression system to produce antitumor biologics.

**Figure 3:**
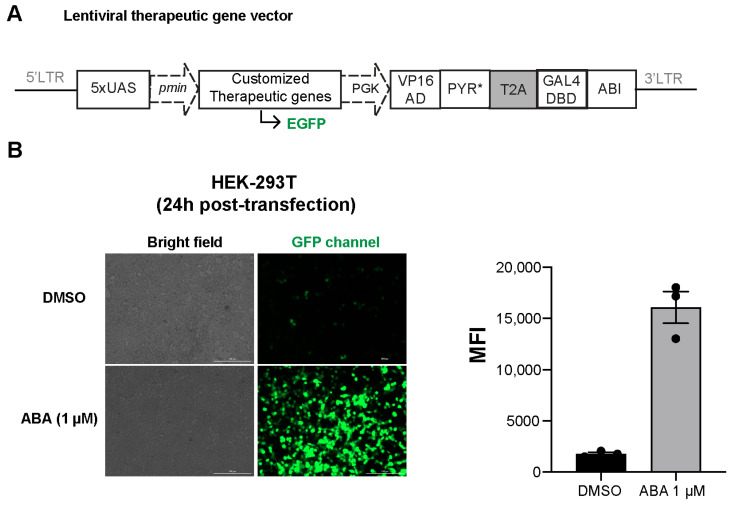
Generation and validation of recombinant lentiviral vector-encoding inducible gene cassette. (**A**) Lentiviral vector of ABA-inducible gene expression system. (**B**) ABA-induced EGFP expression in transfected HEK-293T cells. Cells were treated with 0.5% DMSO or 1 µM ABA, and the induced EGFP expression was observed under a fluorescence microscope after 24 h. The scale bar is 200 µm. Mean fluorescence intensity (MFI) was calculated from 3 random areas in a well using microscope-integrated cellular analysis tool. Error bars are mean ± S.E.M.

Next, we constructed the inducible gene expression system in the lentiviral vector for more effective transgene delivery in T cells. A single lentiviral vector was constructed by combining the split transcriptional activator and the inducible gene expression unit. The ABA-responsive split transcriptional activator was cloned into a second generation pLV lentiviral-expressing vector (N103) downstream of the PGK promoter. The 5x upstream-activating sequence (UAS) response element with a minimal CMV promoter controlling the expression of therapeutic genes were placed before the PGK promoter in the same vector. The whole transgene was flanked by the two long terminal repeat (LTR) elements (Figure 3A). To validate the lentiviral vector design of the inducible therapeutic gene circuit, we constructed the inducible EGFP reporter gene version (N103-iEGFP) to monitor the ABA-inducible EGFP expression using a fluorescence microscope. The N103-iEGFP was transiently transfected into HEK-293T cells, and the cells were treated with or without ABA. At 24 h post-transfection, we observed EGFP expression upon ABA treatment (Figure 3B), indicating the successful construction of the lentiviral vectors.

### 2.2. ABA-Induced Expression and Secretion of Inflammatory Cytokine Interleukine-12 in Human Jurkat T Cells

We next examined whether T cells could be engineered to secrete customized cytokines capable of modulating immune responses. Long-term persistence and expansion of CAR-engineered T cells are required to effectively combat cancer. To overcome tumor suppressive effects, strategies to potentiate the therapeutic activities of CAR-T cells by engineering CAR-T cells that produce non-native cytokines upon tumor antigen engagement have been employed [5,11]. Our strategy, in contrast, was to design CAR-expressing T cells that can drive custom therapeutic responses in an antigen-independent manner through a cancer signal-sensing approach.

**Figure 4:**
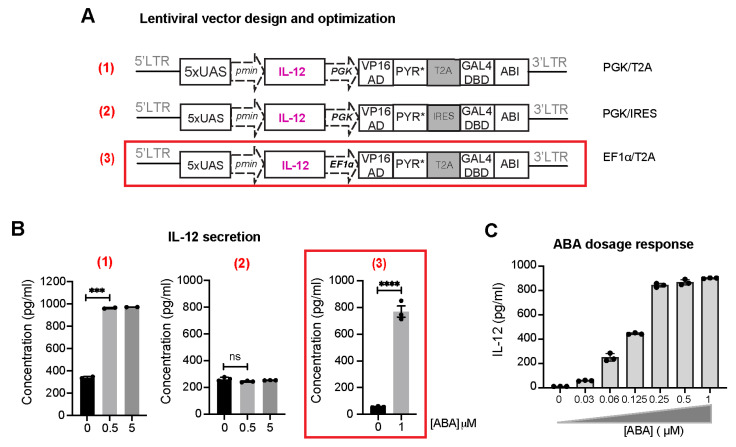
ABA-induced expression of pro-inflammatory cytokine IL-12 in engineered Jurkat T cells. (**A**) Three different configurations of lentiviral vectors were tested. The best construct (3) is outlined in red and used in the following studies. (**B**) Jurkat T cells were then transduced with viruses encoding different inducible IL-12 (iIL-12) vectors and treated with DMSO or ABA at indicated concentrations for 24 h. IL-12 released in the cell culture supernatant was quantified by ELISA. Data are shown as mean ± S.E.M from 3 technical triplicates. **** *p* ≤ 0.0001, *** *p* ≤ 0.001, ns, *p* ≥ 0.05, two-tailed Student *t*-test. (**C**) Dosage response of inducible IL-12 in engineered Jurkat T cells with varying ABA concentrations.

As a hallmark of inflammatory cytokines, interleukin 12 (IL-12), a heterodimeric protein composed of p35 and p40 subunits, plays an important role in bridging innate and adaptive immunity. IL-12 is mainly produced by innate immune cells such as macrophages, dendritic cells, and neutrophils, and has been known as a critical regulator of cell-mediated immunity [23]. Various pre-clinical models have demonstrated that IL-12 modulates antitumor responses at various levels, for example, through the induction of T_H_1 differentiation [24], sustaining survival and re-activation of CD4-T cells [25], and enhancing cytolytic activity of NK cells and CD8 T cells [26]. Therefore, local production of this therapeutic agent will activate innate immune cells, which in turn will maximize the antitumor response in the targeted tumor lesion while avoiding substantial systemic toxicities associated with the high serum level of IL-12 observed in clinical trials [27]. With that in mind, we generated a lentiviral vector for inducible IL-12 expression and secretion upon ABA treatment by replacing EGFP from the N103-iEGFP with the IL-12 gene [11] (i.e., PGK/T2A in Figure 4A). We explored different ways of designing the constructs to obtain the best signal-to-noise induction of the therapeutic gene expression. In one version, the 2A sequence was replaced by an internal ribosome entry site (IRES) and still driven by the PGK promoter (i.e., PGK/IRES, Figure 4A). The 2A-linked genes were expected be expressed at an equal level, while the IRES-mediated bicistronic vector expressed the proteins at different levels, with a lower expression level in the IRES-driven gene [28]. Additionally, the 2A elements are much shorter than IRES, with only 60 to 80 base pairs, which should increase the viral packaging efficiency. Another version was made by replacing PGK with the human elongation factor-1 alpha (EF1α) promoter (i.e., EF1α/T2A, Figure 4A), which is commonly used in viral vector expression systems. We tested the ABA CIP-controlled inducibility in the secretion of IL-12 in human Jurkat T cells using the ELISA assay. These lentiviral vectors were transiently transfected into HEK-293T cells along with two other helper plasmids (i.e., packaging and envelope) to generate lentiviruses, which were then used to infect Jurkat T cells. At day 4 post-transduction, transduced Jurkat cells were treated with or without ABA for 24 h and the amount of IL-12 secreted in the cell culture media was quantified via ELISA. Comparing these three designs, we observed that the PGK/IRES version failed to induce IL-12 production upon ABA treatment as compared to the PGK/T2A version under the control of the same promoter (Figure 4B), which may be due to the differential expression of the two split transcriptional activator components in the IRES case. The EF1α/T2A version resulted in the most optimal inducibility with minimal basal transcriptional activity, which we identified as the most ideal design to move forward. The differential effects between using EF1α and PGK promoters indicated that intricate cellular regulation on promoters should be considered for future optimization. Therefore, we adopted this vector configuration for constructing other therapeutic protein expression vectors. Additionally, the ABA-based CIP system has been reported to yield dosage-dependent controls in biological outputs [29]. To examine whether the amount of IL-12 expression is titratable by varying ABA concentrations, Jurkat T cells were transduced with inducible IL-12-encoding EF1α/T2A lentivirus and treated with ABA at different concentrations ranging from 0 to 1000 nM for 24 h before quantifying IL-12 in the media using ELISA. We observed that there was a clear ABA-dependent dosage response with a maximum induction at around 250 nM (Figure 4C). This system can potentially provide an important tool to fine-tune the secreted IL-12 dosage to achieve substantial efficacy without severe adverse effects.

Next, we tested the efficiency of generating Jurkat T cells with both CAR and ABA-inducible therapeutic gene expression lentiviral vectors. We first constructed a second generation CAR vector targeting human epidermal growth factor receptor 2 (HER2/ErbB2). HER2 is a well-characterized antigen and therapeutic target expressed in a variety of tumors ranging from breast cancers and ovarian cancers to osterosacomas, and also expressed at low levels in normal tissues [30,31,32,33,34]. The HER2-CAR lentiviral vector comprises a HER2-targeted scFv sequence derived from the humanized mAb trastuzumab (4D5-5 clone) [34], a hinge and transmembrane domain of human CD8α molecules, a costimulatory 4-1BB, and intracellular signaling CD3ζ domains, driven by the EF1α promoter (Figure S1A). The CAR also contains an N-terminal CD8α signal peptide responsible for membrane targeting, and a Myc tag at the N-terminus for the quantification of surface expression of CAR. We first evaluated the surface expression of HER2-CAR on Jurkat T cells by transducing Jurkat cells with the HER2-CAR lentiviral vector and quantified the HER2-CAR expression through the Myc tag using flow cytometry. We confirmed the effective surface expression of HER2-CAR on Jurkat T cells on day 4 post-transduction (Figure S1B). We also validated the function of HER2-CAR by stimulating anti-HER2 CAR-Jurkat cells with target cell lines expressing different levels of HER2 (Figure S1C). The activation of the engineered Jurkat cells under each co-incubation condition was determined via the surface expression of activation maker-CD69 and the secretion of IL-2 cytokine (a critical and early landmark of T-cell activation). We confirmed that the HER2-CAR-expressing Jurkat T cells were only activated in the presence of HER2-expressing cells (Figure S1D).

**Figure 5:**
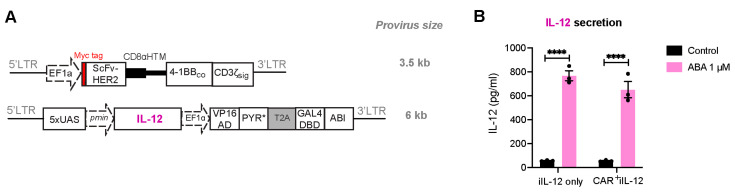
ABA-induced expression of pro-inflammatory cytokine IL-12 in CAR^+^iIL-12 Jurkat T cells. (**A**) Lentiviral vectors encoding HER2-CAR and ABA-inducible IL-12 expression cassette. (**B**) The comparison of IL-12 expression in Jurkat T cells transduced with a single virus encoding iIL-12 only or two viruses encoding both iIL-2 and CAR (CAR^+^ iIL-12) genes. Cells were treated with DMSO or ABA (1 μM), and IL-12 secretion was quantified by ELISA at 24 h post-treatment. Data are shown as mean ± S.E.M from 3 technical replicates. **** *p* ≤ 0.0001, two-tailed Student *t*-test.

Next, the HER2-CAR and ABA-inducible IL-12 expression circuits were delivered into Jurkat T cells by two individual lentiviral vectors (Figure 5A). The integrated provirus sequence between the two LTRs is about 3.5 kb and 6 kb for HER2-CAR and inducible IL-12 vector, respectively, which is within the viral packaging limit. Transduced Jurkat T cells either expressed the inducible IL-12 gene only (iIL-12) or the inducible IL-12 gene plus HER2-CAR (CAR^+^iIL-12) for 4 days, and were then treated with ABA for 24 h before the cell culture supernatant was harvested and analyzed for the secretion of IL-12 using ELISA. We observed similar amounts of IL-12 production from both groups, indicating comparable and satisfactory efficiencies in the production of IL-12 even when co-transducing dual constructs (Figure 5B). Furthermore, we examined the expression of HER2-CAR when co-transduced with the IL-12 expression circuits and observed a satisfactory transduction efficiency (Figure S2A).

### 2.3. ABA-Induced Expression and Secretion of Immune Checkpoint (ICP) Inhibitors in Engineered Jurkat T Cells

**Figure 6:**
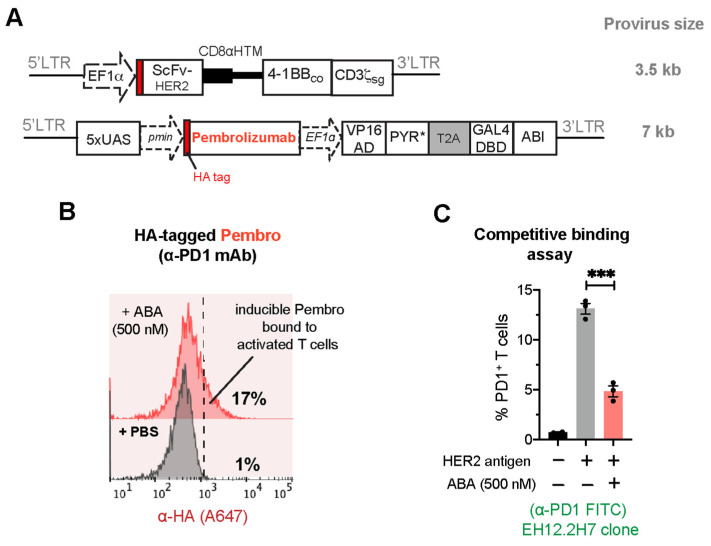
ABA-induced expression of immune checkpoint inhibitor pembrolizumab (α-PD1 mAb) in CAR^+^iPembro Jurkat T cells. (**A**) Lentiviral constructs for HER2-CAR and ABA-inducible pembrolizumab. (**B**) CAR^+^ T cells were co-cultured with MDA-MB-231 cancer cells at 5:1 E:T ratio in the presence of 500 nM ABA or PBS for 24 h. Jurkat T cells were stained for Pembro using A647-conjugated α-HA antibody. The shift in fluorescence signal towards the right indicated the binding of the secreted Pembro to the PD-1 ligand. (**C**) The levels of free PD-1 were detected by staining the cells with commercial FITC-conjugated α-PD1 antibody (clone EH12.2H7) and analyzed via flow cytometry. Data are mean ± S.E.M from 3 biological replicates. *** *p* ≤ 0.001, two-tailed Student *t*-test.

To counteract the negative effect of TME on CAR-T function, combining CAR-T and ICP blockades has been proven an efficacious treatment approach against solid tumors [35,36,37,38]. ICP inhibitors, delivered either through the constitutive expression by CAR-T cells or systemic administration, come with several adverse effects. Toxicities associated with ICP blockades include, but are not limited to, the increased activation of autoreactive T cells due to systemic blocking of ICP pathways. A potential way to circumvent current systemic toxicities while enhancing the effectiveness of ICP inhibitors is to have T cells locally produce these therapeutics in tumors. Amongst the ICP inhibitors developed so far, blocking programmed cell death protein 1 (PD-1) expression on activated T cells using the clinically approved monoclonal antibody pembrolizumab (α-PD1 mAb) has achieved remarkable successes in treating various solid cancers and lymphomas [39,40,41]. PD-1 binds programmed death ligand 1 (PD-L1), which is expressed on many tumor cells, resulting in the suppression of T-cell proliferation and cytokine production and correlating with the expression of “T-cell exhaustion” markers such as LAG-3, TIM-3, and CD160 [42]. Thus, we developed CAR^+^ cells capable of secreting pembrolizumab (hereafter “Pembro”) directed against PD-1 in response to ABA induction. To test the production of Pembro, we used a cell-based flow cytometry binding assay in which the secreted Pembro binds to the target cell surface antigen PD-1, and the bound Pembro can then be analyzed by flow cytometry. Pembro [11] was cloned into the ABA-inducible lentiviral vector, which also contains an HA tag for detection purposes (Figure 6A). Jurkat T cells were transduced by lentiviruses to co-express the HER2-CAR and the inducible Pembro (CAR^+^iPembro), and the transduced cells were co-incubated with HER2-expressing MDA-MB-231 cancer cells, which triggered PD-1 expression on the activated CAR-Jurkat cells. These cells were treated with 500 nM ABA or PBS (as a control) for 24 h, and the secretion and binding of Pembro to PD-1 was then detected using flow cytometry by staining Jurkat cells with fluorophore-conjugated α-HA antibodies. The increase in the HA fluorescence signal indicated the expression and secretion of Pembro and its binding to PD-1 on CAR^+^iPembro Jurkat cells only in the presence of ABA, but not PBS (17% vs. 1%) (Figure 6B). We also confirmed the satisfactory expression of HER2-CAR when co-transduced with the inducible Pembro expression circuits (Figure S2B). To confirm that the secreted Pembro specifically bound to PD-1 on activated Jurkat cells, we performed a competitive binding assay using a commercially available α-PD1 antibody (clone EH12.2H7) to measure the levels of free PD-1 on the cell surface. The commercial α-PD1 antibody has been shown to bind to the same epitope on PD-1 as Pembro [43]; therefore, any binding between Pembro and PD-1 will interfere with the binding of the commercial α-PD1 to PD-1. CAR^+^iPembro cells in the co-culture treated with either ABA or PBS for 24 h were stained for PD-1 using FITC-conjugated α-PD1 antibody (α-PD1 FITC). The percentage of PD-1-positive cells was determined using flow cytometry. As shown in Figure 6C, the percentage of PD1-positive Jurkat cells was significantly lower in the ABA-treated group, suggesting that Pembro was present and occupied PD-1 on the Jurkat cell surface, which blocked the interaction between the commercial α-PD1 antibody and PD-1. It was also observed that CAR^+^iPembro Jurkat cells without co-culturing with HER2^+^ tumor cells did not express PD-1 due to lack of tumor-mediated T-cell activation.

**Figure 7:**
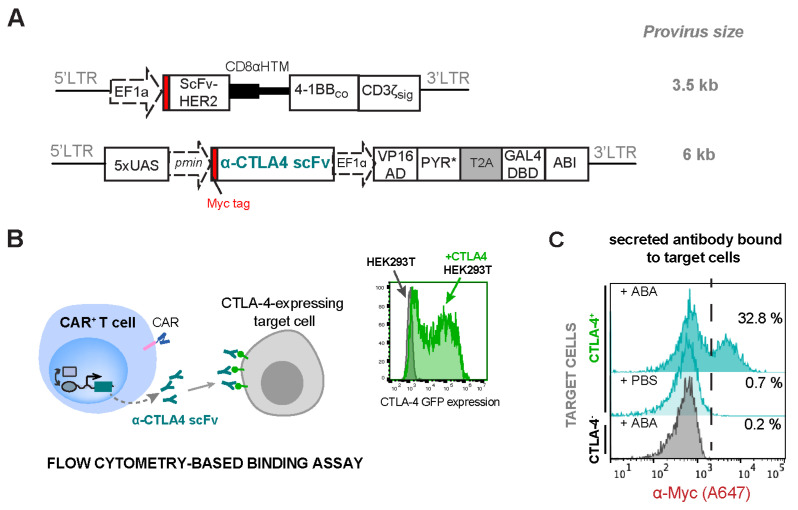
ABA-inducible expression of immune checkpoint blockage α-CTLA4 scFv in CAR-expressing Jurkat T cells. (**A**) Lentiviral constructs for HER2-CAR and ABA-inducible α-CTLA4 scFv. (**B**) The secreted α-CTLA4 scFv in the cell culture supernatant was detected using flow cytometry-based binding assay and HEK-293T target cells overexpressing the CTLA4 ligand. The scFv-ligand binding was followed by staining target cells with A647-conjugated α-Myc antibody for detection of the bound scFv to target cells. (**C**) Flow cytometry data showing the induced expression and secretion of α-CTLA4 scFv upon the addition of 500 nM ABA or PBS and selective binding of the scFv to CTLA-4^+^ cells. The shown histogram is a representative of 3 biological replicates.

Another ICP inhibitor of interest is α-CTLA4 antibodies that target the CTLA-4/B7 checkpoint pathway. CTLA4 checkpoint molecules are primarily expressed by T cells and compete with CD28 for the binding of the B7-family costimulatory molecules [44]. This competitive binding can block the costimulatory signal required for T-cell activation, resulting in the inhibition of T-cell proliferation and survival. To express iCTLA4 scFv, a Myc-tagged scFv version of α-CTLA4 monoclonal antibodies [11] (derived from ticilimumab) was cloned into the ABA-inducible therapeutic gene vector (Figure 7A). Jurkat T cells transduced to express both HER2-CAR and the iCTLA4 scFv (CAR^+^iCTLA4 scFv) were treated with or without 500 nM ABA for 24 h to induce the expression and secretion of α-CTLA4 scFv, which was detected by a flow cytometry-based binding assay. We overexpressed CTLA4 antigen on HEK-293T cells so that these cells can serve as “target cells” for the binding assay (Figure 7B). Cell culture supernatant containing the secreted antibodies from CAR^+^iCTLA4 scFv Jurkat cells was incubated with surface CTLA4 antigen-positive or negative target HEK-293T cells, followed by staining the target cells with Alexa Flour 647-conjugated α-Myc to detect bound antibodies. The fluorescence intensity correlates with the amount of α-CTLA4 scFv produced by the CAR^+^iCTLA4 scFv Jurkat cells. As shown in Figure 7C, CAR^+^iCTLA4 scFv Jurkat cells produced a high level of the α-CTLA-4 scFv when ABA was added (32.8% vs. 0.7%) as detected on CTLA4^+^ target cells, and the α-CTLA-4 scFv selectively bound only to antigen-expressing cells (32.8% vs. 0.2%). We also confirmed the efficient expression of HER2-CAR when co-transduced with the inducible α-CTLA4 scFv expression circuits (Figure S2C). Taken together, these data show that ABA can effectively trigger engineered CAR^+^iCTLA4 scFv Jurkat T cells to express different immune checkpoint inhibitors.

### 2.4. Jurkat T Cells Equipped with CAR and Conditional Gene Circuit Can Respond to β-Gal to Drive the Expression and Secretion of Customized Therapeutics

**Figure 8:**
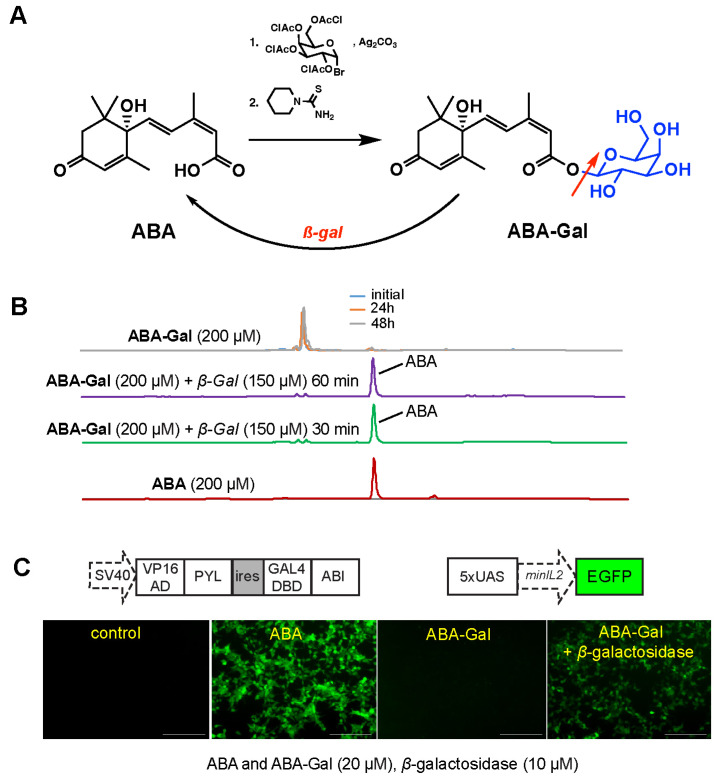
Synthesis and characterization of ABA prodrug (ABA-Gal) for *β*-galactosidase (*β*-gal). (**A**) Synthesis and the activation of ABA-Gal prodrug. Red arrow indicates cleavage of the prodrug by *β*-gal. (**B**) The stability of ABA-Gal and its generation of ABA by *β*-gal were analyzed using HPLC. Prodrug at 200 μM was incubated with *β*-gal at the indicated concentration in PBS buffer (pH 7.4) at 37 °C for different time points. (**C**) HEK-293T EGFP reporter cells responded to *β*-gal when treated with ABA-Gal, leading to EGFP expression. Reporter cells were treated with 20 μM ABA or 20 μM prodrug with and without 10 μM *β*-gal. Treated cells were incubated for 24 h, and EGFP expression was observed under a fluorescence microscope. The scale bar is 200 µm.

The unique design in our strategy is to use cancer-associated signals to induce local activation of the inducer small molecule ABA, which subsequently triggers the targeted production and release of in situ therapeutics and immunomodulators that are expected to reduce systemic toxicities and enhance the therapeutic efficacy of the engineered T cells. To achieve this goal, we designed and synthesized a caged ABA prodrug (ABA-Gal) that is sensitive to tumor-associated *β*-gal as a proof-of-principle study (Figure 8A). D-ABA-Gal was previously synthesized to study plant hormone profiling [45]. We synthesized ABA-Gal following the reported procedures [45] and used high performance liquid chromatography to assess the chemical stability and reactivity of the ABA-Gal compound toward the recombinant *β*-galactosidase enzyme in vitro. A molarity of 200 μM ABA-Gal in PBS was incubated at 37 °C for up to 2 days, followed by HPLC analyses. We observed that the prodrug was stable in buffer during the observation period. *β*-gal-induced cleavage of ABA-Gal was also confirmed by incubating 200 μM ABA-Gal with 150 μM recombinant *β*-gal at 37 °C. We found that the compound was quickly uncaged by *β*-gal to generate ABA within 30 min (Figure 8B). Using HEK293T-EGFP reporter cell line [16], which can respond to ABA and express EGFP, we observed that ABA-Gal was stable within the 24 h observation period, while the addition of recombinant *β*-gal resulted in the induction of EGFP expression (Figure 8C), indicating the release of functional ABA in the cell culture.

**Figure 9:**
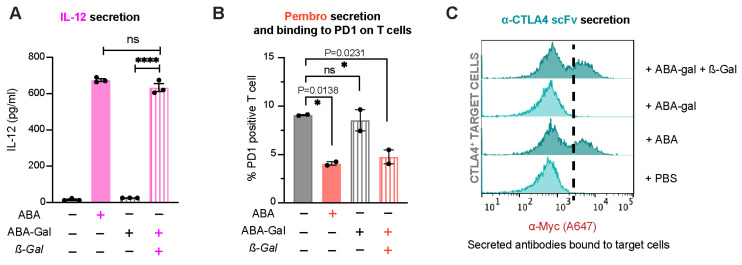
TME-gated expression and secretion of customized biologics in engineered Jurkat T cells. (**A**) The secretion of IL-12 by CAR^+^iIL12 cells at 15 h post-drug treatment, as detected via ELISA. Data are shown as mean ± S.E.M from 3 technical triplicates. **** *p* ≤ 0.0001, ns, *p* ≥ 0.05, two-tailed Student *t*-test. (**B**) Competitive binding assay showing % PD1-positive Jurkat T cells in a 24 h co-culture of CAR^+^iPembro cells and HER2-expressing cancer cell line MDA-MB-231. Data are mean ± S.E.M from 2 biological replicates. * *p* < 0.05, ns, *p* ≥ 0.05, one-way ANOVA with Dunnett multiple comparisons test. (**C**) The secretion of α-CTLA4 scFv by CAR^+^iCTLA4 scFv and the binding of scFv to CTLA4-expressing HEK-293T target cells, followed by staining target cells with A647-conjugated anti-Myc antibody and detected via flow cytometry. The histograms shown are representative of 2 biological replicates.

Next, we examined if this prodrug strategy can be integrated into our platform that allows engineered T cells to analyze and respond to *β*-gal and activate custom immunomodulating programs. Jurkat T cells were transduced to express HER2-CAR along with different ABA-inducible gene cassettes, as described above, followed by the treatment with 500 nM ABA or 500 nM ABA-Gal with or without 100 nM *β*-gal for indicated time periods. Using the same assays developed for testing each therapeutic protein, as described above, we observed that the production and secretion of IL-12, Pembro or α-CTLA4 scFv were only detected when cells were treated with ABA or with ABA-Gal plus *β*-gal, suggesting that *β*-gal reliably activated ABA-Gal to produce ABA, which in turn triggered the production of therapeutic proteins (Figure 9). These data established that *β*-gal can trigger engineered Jurkat T cells to express different therapeutic and immunomodulatory modalities.

## 3. Materials and Methods

### 3.1. Synthesis of ABA-Gal

All starting reagents and solvents were directly used without any further purifications and air-sensitive reactions performed in an argon atmosphere. The progress of reactions was examined with the aid of a thin layer chromatography (TLC) technique consisting of a silica gel plate precoated with fluorescent indicator (254 nm) under UV light (260 nm). Purification of the compounds was carried out via column chromatography over silica gel 60–120 mesh (Sigma-Aldrich, St. Louis, MO, USA), stationary phase. All ^1^H and ^13^C NMR spectra were recorded on a Bruker Avance III-HD 500 NMR spectrometer instrument, whereas high-resolution mass spectra (HRMS) were recorded on a Waters Micromass mass spectrometer with electrospray ionization probe using positive ion mode. The chemical shifts and apparent coupling constant values were reported in parts per million (ppm) and Hertz, respectively. These chemical shifts were referenced from the TMS signal or deuterated residual solvents: CDCl_3_ (7.26 ppm), CD_3_OD (3.31 ppm), DMSO-*d6* (2.5 ppm), and D_2_O (4.79 ppm) for ^1^H NMR spectra and CDCl_3_ (77.1 ppm), CD_3_OD (49.0 ppm), and DMSO-*d6* (39.5 ppm) for ^13^C NMR spectra. The ^1^H NMR spin coupling multiplicities are listed such as s (singlet), d (doublet), t (triplet), dd (doublet of doublets), and (q) quintet or m (multiplet and overlapping spin signal). ABA-Gal was synthesized following the reported procedures [45].

*R_f_* = 0.3 (DCM:MeOH = 9:1). ^1^H NMR (500 MHz, DMSO-d_6_): δ 7.73 (d, *J* = 15.8, 1H), 6.35 (s, *J* = 15.9, 1H), 5.84 (s, 1H), 5.76 (s, 1H), 5.33 (d, *J* = 7.9 Hz, 1H), 5.29 (s, 1H), 5.07 (s, 1H), 4.88 (s, 1H), 4.67 (s, 1H), 4.55 (s, 1H), 3.68 (s, 1H), 3.51–3.34 (m, 5H), 2.11 (d, *J* = 16.7, 1H) 2.04 (s, 3H), 1.8 (s, 3H), 1.62 (s, 1H), 0.96 (s, 3H), 0.93 (s, 3H); ^13^C NMR (125 MHz, DMSO-d_6_): δ 197.7, 164.4, 163.5, 152.9, 139.6, 127.4, 127.5, 116.9, 94.8, 78.9, 76.6, 73.7, 69.9, 68.4, 60.6, 49.7, 41.8, 24.6, 23.6, 21.4, 19.3; HRMS (ESI): calcd for C_21_H_30_O_9_: [M + Na]^+^, 449.1782; found: [M + Na]^+^, 449.1787 (Δm = +0.0005 and error = +1.1 ppm).

### 3.2. Chemical Stability and Reactivity of ABA-Gal towards β-Gal In Vitro Using HPLC

In vitro stability testing was carried out by incubating ABA-Gal in PBS buffer (pH 7.4) up to 48 h at 37 °C. HPLC data were obtained at 0 h, 24 h, and 48 h. The chromatograms were collected using a Dionex-UltiMate 3000 LC System with Acclaim 120 Å, C18, 3 μm analytical (4.6 × 100 mm) column (Thermofisher, Waltham, MA, USA). Reactivity of ABA-Gal towards *β*-gal (Sigma-Aldrich, St. Louis, MO, USA) was performed by incubating 200 μM ABA-Gal with 150 μM *β*-gal enzymes at 37 °C for different time points. Then, the reaction mixture was quickly injected into HPLC and analyzed.

All HPLC analyses were run at RT and monitored at 260 nm. A gradient of acetonitrile in 0.1%TFA and water containing 0.1% TFA were used at a flow rate of 0.750 mL/min. Acetonitrile was increased from 5% to 75% in 15 min and kept constant for 3 min. The total running time was 20 min. HPLC chromatograms were acquired using a Dionex-UltiMate 3000 LC System with Acclaim 120 Å, C18, 3 μm analytical (4.6 × 100 mm) column.

### 3.3. Cellular Stability of ABA-Gal and Cleavage Activity of β-Gal in HEK-293T Cells

Stock concentrations of ABA and ABA prodrug were prepared in DMSO and further diluted in PBS for subsequent cellular experiments.

To test the cellular stability and reactivity of ABA-Gal, EGFP-expressing cells (HEK 293-GFP) were seeded in triplicate in 24-well plates (10^6^ cell/mL) at a final volume of 500 μL. The cells were treated with either ABA, ABA-Gal, or ABA-Gal plus *β*-gal at indicated concentrations, 24 h after plating. Following another 24 h, live cells were imaged with an Axio Observer (Zeiss, White Plains, NY, USA) fluorescence microscope under GFP channel at the UNM Chemistry department facility (Albuquerque, NM, USA).

### 3.4. Cell Lines and Culture

Human embryonic kidney cells (HEK-293T) were obtained from Dr. Alex Huang’s lab CWRU School of Medicine (Cleveland, OH, USA). The HEK-293T cell line was also used for lentiviral packaging and preparation. The reporter cell line EGFP-expressing human embryonic kidney (HEK-293T-EGFP) was generated and maintained in our laboratory. Human breast adenocarcinoma MDA-MB-231 was maintained in our lab. These cells were grown in Dulbecco’s modified Eagle’s medium (DMEM) (Gibco, Waltham, MA, USA) supplemented with 10% (*v*/*v*) heat-inactivated fetal bovine serum (FBS, Omega Scientific, Tarzana, CA, USA), 5% (*v*/*v*) GlutaMAX (Life Technologies, Carlsbad, CA, USA), and 100 IU/mL penicillin/streptomycin (Life Technologies, MA, USA). Jurkat T cells, clone E6-1, were purchased from American Type Culture Collection (ATCC) (Manassas, VA, USA) and cultured in complete RPMI-1640 containing 2 mM L-glutamine, 10 mM HEPES, 1 mM sodium pyruvate, 4500 mg/L glucose, and 1500 mg/L sodium bicarbonate supplemented with 10% heat-inactivated fetal bovine serum (FBS) and 100 IU/mL penicillin/streptomycin. All cultures were maintained at 37 °C in a humidified incubator containing 5% CO_2_. Cells were confirmed to be mycoplasma-free using the MycoAlert™ mycoplasma detection kit (Lonza, Walkersville, MD, USA).

### 3.5. Plasmid Construction

Construction of the ABA-split transcriptional activator construct sv40-VP16-PYR*-ires/T2A-Gal4DBD-ABI_D134A_ has been described previously [16]. The ABA-inducible customized therapeutic constructs were made by inserting different therapeutic genes under the control of a minimal IL-2 (or minimal CMV) gene promoter and five copies of Gal4 DNA binding sites (CGGAGTACTGTCCTCCGAG). The therapeutic genes used in this study are tumor necrosis factor-related apoptosis-inducing ligand (TRAIL), interleukin-12, immune checkpoint inhibitors pembrolizumab mAb, and α-CTLA-4 scFv. Sources of the therapeutic genes are listed in Table S1. The conventional CAR construct targeting HER2 antigen comprising CD8α signal sequence-Myc tag-αHer2 scFv-CD8α_Hinge_-CD8α_TM_-41BB-CD3ς was designed and synthesized as gBlock gene fragments (IDT). The vector components are described in Table S1. The genes were cloned into a 2nd generation N103 lentiviral transfer vector, under the control of an EF-1α promoter using In-fusion cloning (Takara Bio, San Jose, CA, USA). The expression cassettes for the ABA-split transcriptional activator (VP*2GA) and therapeutic gene were cloned into a bidirectional N103 lentiviral vector. The vector containing the VP*2GA was placed under the control of PGK or EF1α promoter while the response element (5xUAS) controlling the expression of customized therapeutic proteins (described previously) was inserted upstream of PGK or EF-1α promoter, under the control of a minimal CMV promoter. The wildtype lentiviral genome is ~9.2 kb; therefore, all transgenes were made within the viral packing limit (3–9 kb) for efficient viral production.

All plasmid constructs were amplified using a Stbl3 chemically competent *E. coli* strain and purified using the Endo-free Qiagen Miniprep kit. DNA sequences of the final constructs were confirmed by Sanger sequencing (Genewiz, South Plainfield, NJ, USA). Plasmid concentration and purity (A 260/280 = 1.88–1.90) were measured by a Nanodrop spectrophotometer (Thermofisher).

### 3.6. Production of Lentivirus

Lentivirus was produced by transient transfection into HEK-293T cells. One day before transfection, cells were plated at a density of 7 × 10^6^ cells per 150 mm diameter tissue culture plate in 30 mL of DMEM supplemented with 10% (*v*/*v*) FBS and 5% (*v*/*v*) GlutaMAX (Life Technologies, MA, USA), and cultured at 37 °C in a 5% CO_2_ incubator. Cells were transfected with a 2nd generation packaging plasmids system (gifted by Crabtree’s lab, CA, USA) at ~80% confluence. The transfection complex was prepared in two separate 1.7 mL tubes. A mixture of 6 μg of transfer plasmid containing corresponding GOI, 1.5 μg of ps.PAX2 (packaging plasmid), and 4.5 μg of pMD2.G (envelope plasmid) were mixed in 120 μL of Opti-MEM (Gibco) (tube A), and 36 μL of polyethyleneimine (PEI) transfection reagent (Polysciences, Warrington, PA, USA) were diluted in 444 μL of Opti-MEM (tube B). The contents of tube A and tube B alone were incubated at RT for 5 min before combining into a single tube, followed by another 20 min incubation. The transfection complex was added into the cells, and media was replaced with complete DMEM 6 h post-transfection. The culture supernatant containing viral particles was harvested at 48 h post-transfection, and filtered through a 0.45 μm syringe filter (VWR, Secaucus, NJ, USA). Viral particles were concentrated by ultracentrifugation at 100,000× *g* at 4 °C for 100 min with a Sorval LYNX T29-8x50 Rotor (Thermofisher) and resuspended in 500 μL of complete RPMI medium. Stock viral supernatant was either used directly or aliquoted and stored in a −80 °C freezer until ready for use.

### 3.7. Transduction of Jurkat T Cells

For transduction of Jurkat T cells, an appropriate amount of the pooled virus was added to Jurkat T cells (10^6^ cells/mL) in the presence of 8 μg/mL polybrene (Santa Cruz Biotech, TX, USA) in a non-treated tissue culture 24-well plate, and the plate was centrifuged at 1000× *g* at 33 °C for 2 h (spinoculation). The media was changed to complete fresh RPMI and the cells were cultured at 37 °C and 5% CO_2_ for at least 3 days before subsequent experiments. Fresh media was added to the well frequently to maintain a cell density of 0.5–2 × 10^6^ cells/mL.

### 3.8. Apoptosis Assay and Quantification of Secreted TRAIL

For sTRAIL apoptotic assays, HEK-293T cells were transiently transfected to express the new potent ABA-responsive split transcriptional activator and 5xGal4 response elements controlling the expression of sTRAIL. Cell culture supernatant containing sTRAIL was collected after 24 h and treated with either 1μM ABA or mock DMSO and incubated with TRAIL-sensitive MDA-MD-231 cancer cell lines for another 24 h. Cell viability and apoptosis were accessed using the Dead Cell Apoptosis Kit with Annexin V-FITC and PI for flow cytometry according to the manufacturer’s protocol. Briefly, cancer cells were harvested by trypsinization at 24 h after drug treatment and were washed twice with cold PBS. Then, 10^6^ cells, suspended in Annexin V binding buffer, were incubated with propidium iodide and FITC-Annexin V to a final concentration of 1 μg/mL and a 1:20 volume ratio, respectively, for 15 min at room temperature in the dark. Subsequently, 400 μL of annexin-binding buffer was added, and the stained cells were immediately analyzed using a flow BD Accuri™ C6 cytometer (BD Biosciences, San Jose, CA, USA). The concentration of secreted TRAIL in the supernatant was determined via the human TRAIL PicoKine^TM^ ELISA kit (Boster Bio, Pleasanton, CA, USA).

### 3.9. Signal-Induced IL-12 Production and Quantification

Human Jurkat T cells were transduced with either a therapeutic-encoding vector alone or with conventional CAR and treated with small-molecule or small-molecule plus TME signals to induce the production and secretion of IL-12 into the cell culture media. At the indicated time point, the cell culture supernatant was harvested and stored for analysis at −80 °C or analyzed directly using an ELISA kit (Biolegend, San Diego, CA, USA). One day before the assay, a 96-well ELISA plate was coated with human IL-12 specific capture antibody and stored overnight at 4 °C. After 1 h of blocking plate, the ELISA was performed according to the manufacturer’s instructions. Human IL-12 standards were used to generate a standard curve of range 0–500 pg/mL. Absorbance was recorded at 450 nm and 570 nm on a Spectramax i3X microplate reader (CWRU Core Facilities, Cleveland, OH, USA). Data were analyzed with GraphPad Prism software version 8.4.0. 

### 3.10. Signal-Induced Immune Checkpoint Antibodies Production and Quantification

Flow cytometry-based binding assay was used to detect secreted immune checkpoint antibodies in the cell culture supernatant. Jurkat T cells were transduced to inducibly express ICP antibodies including pembrolizumab or α-CTLA-4 scFv. Transduced cells were treated with ABA or PBS (as control) for 24 h to induce the production of antibodies. Cell culture supernatant containing the secreted antibody was harvested and stored at −80 °C for analysis. To detect secreted α-CTLA-4 scFv, HEK-293T cells overexpressing human CTLA-4 were used as “target cells”. Target cells were incubated with culture supernatant containing secreted α-CTLA-4 scFv for 1 h at RT, followed by staining with fluorescence-conjugated α-Myc antibodies to detect bound antibodies. Then, antibody-labeled target cells were analyzed by flow cytometry to quantify the fluorescence intensity, which correlated with the amount of antibody produced by CAR^+^ T cells.

Secretion and binding of pembrolizumab (α-PD-1) to PD-1 receptors on T-cell surfaces were accessed using competitive binding assay. CAR^+^ T cells were incubated with HER2/PDL1-positive target at E:T = 5:1, and small-molecule or prodrug plus-activating signal was added to the cell culture. At 24 h, T cells were harvested and stained with FITC-conjugated α-PD1 antibody (Biolegend, dilution 1:100) to measure the percentage of positive PD1-T cells, which correlated with the amount of free PD-1 receptor. Direct detection of pembrolizumab was also performed using A647-conjugated anti-HA antibody (CST, dilution 1:50) (Danvers, MA, USA). Data acquisition was performed with a BD Accuri™ C6 Plus Flow Cytometer (BD Biosciences) and analyzed with FlowJo software (v10, TreeStar, Woodburn, OR, USA).

### 3.11. Flow Cytometric Analysis

For flow cytometric analysis, cells were washed and prepared in ice-cold FACS buffer (1x DPBS, 0.5% FBS, 2.5 mM EDTA). Surface expression of HER2 antigen on various target cell lines was confirmed by analysis of cells stained with PE-conjugated anti-human CD340 (Biolegend, dilution 1:100). Expression of CAR was quantified by staining transduced T cells with A488-conjugated anti-Myc antibody (CST, dilution 1:50). Surface expression of CD69 was detected by staining activated T cells with APC-conjugated anti-human CD69 (Biolegend, dilution 1:100). Cells were incubated with fluorophore-conjugated antibodies for 20 min at 4 °C in the dark and washed twice before resuspension with FACS buffer. The percentage of positive cells was calculated by gating on the live cells (based on side and forward scatters), then singlets (based on area vs. height), followed by gating on the fluorescent-labeled population. Data acquisitions were performed with the BD Accuri™ C6 Plus Flow Cytometer (BD Biosciences) and analyzed with FlowJo software (v10, TreeStar).

### 3.12. Statistical Analysis

Data are represented as mean ± standard deviation (SD) or standard error (S.E.M). Statistical significance was calculated in GraphPad Prism (GraphPad software, version 8.4.0, San Diego, CA, USA) using two-tailed unpaired Student’s *t*-test for two independent groups and ANOVA for multiple groups comparison.

## 4. Conclusions

In this study, we presented a proof-of-concept demonstration that signal-sensing ABA prodrug CIP strategy can be incorporated with CAR expression in T cells. This strategy was designed to combine conventional CAR with the ABA-inducible therapeutic gene circuit to achieve in situ site-specific production of various therapeutics, which when applied in CAR-T therapies can contribute to enhance the activity and persistence of CAR-T cells while minimizing the toxicity of systemic administration. Using the Jurkat T cell line, we showed how human Jurkat T cells engineered with CAR and the conditional gene circuit can respond to a unique cancer-associated signal (i.e., *β*-*gal*) in vitro to activate the production of different therapeutic proteins and immunomodulators that can potentially supplement the native response capabilities of CAR-T cells. We have previously shown the feasibility of caging ABA with various sensor units that can be activated by various chosen cellular or disease-related stimuli [13,15,16]. The customizability of our design could also enable the selection of therapeutics for different disease situations as well as the response to various cancer signals that activate corresponding ABA prodrugs.

In the current study, we used human Jurkat T cells as a CAR-T model, which are widely used by others for conducting in vitro CAR testing [46,47]; this allowed us to test the cancer signal-inducible therapeutic gene expression in the CAR-T setting. However, to further advance this approach toward therapeutic applications, this system needs to be further investigated and characterized using CAR produced by relevant human primary T cells and tested in vivo. We expect that further optimization of the gene circuit construct in combination with CAR will be required to achieve optimal outcomes in primary T cells and in vivo. Previous studies have shown that ABA can be used in mouse models and is generally well tolerated by mice [12,48], which should support the feasibility of using the ABA-induced therapeutic gene system to enhance CAR-T functions in xenograft mouse cancer models. Although it has also been reported that ABA can be pro-inflammatory and increase insulin levels [49,50], the ABA and prodrug concentrations used in our studies are much lower than the concentrations reported to induce these effects. Furthermore, the dosage response of the ABA-CIP system potentially enables the fine-tuning of the induced effects while minimizing potential side effects [12,14]. We believe that this novel strategy utilizing ABA prodrug/CIP technology represents a promising approach that can be applied to develop next-generation cancer-sensitized CAR-T cells for cancer immunotherapies that may offer improved efficacy and safety profiles.

## 5. Patents

H.T.X.N. and F.-S.L. are listed as inventors on a patent application describing the technology used in this manuscript. The other authors have no financial conflicts of interest.

## Data Availability

The original contributions presented in the study are included in the article/Supplementary Material; further inquiries can be directed to the corresponding author.

## Supplementary Materials

The following supporting information can be downloaded at: https://www.mdpi.com/article/10.3390/ijms251910568/s1.
