# Supplementary material for: A Customizable Platform to Integrate CAR and Conditional Expression of Immunotherapeutics in T Cells
Source: Int J Mol Sci. 2024 Sep 30;25(19):10568. doi: 10.3390/ijms251910568 (PMC11476998; doi:10.3390/ijms251910568)
Supplement: Supplementary file 1 [file ijms-25-10568-s001.zip › ijms-3172502-supplementary.pdf]

A customizable platform to integrate CAR and conditional expression of immunotherapeutics in T cells

Huong T. X. Nguyen[1], Yabin Song[2], Satendra Kumar[1], Fu-Sen Liang[1*]

[1]Department of Chemistry, Case Western Reserve University, Cleveland, OH, United States
[2]Department of Chemistry and Chemical Biology, University of New Mexico, Albuquerque, NM, United States

Supplementary Information

Supplementary Figures

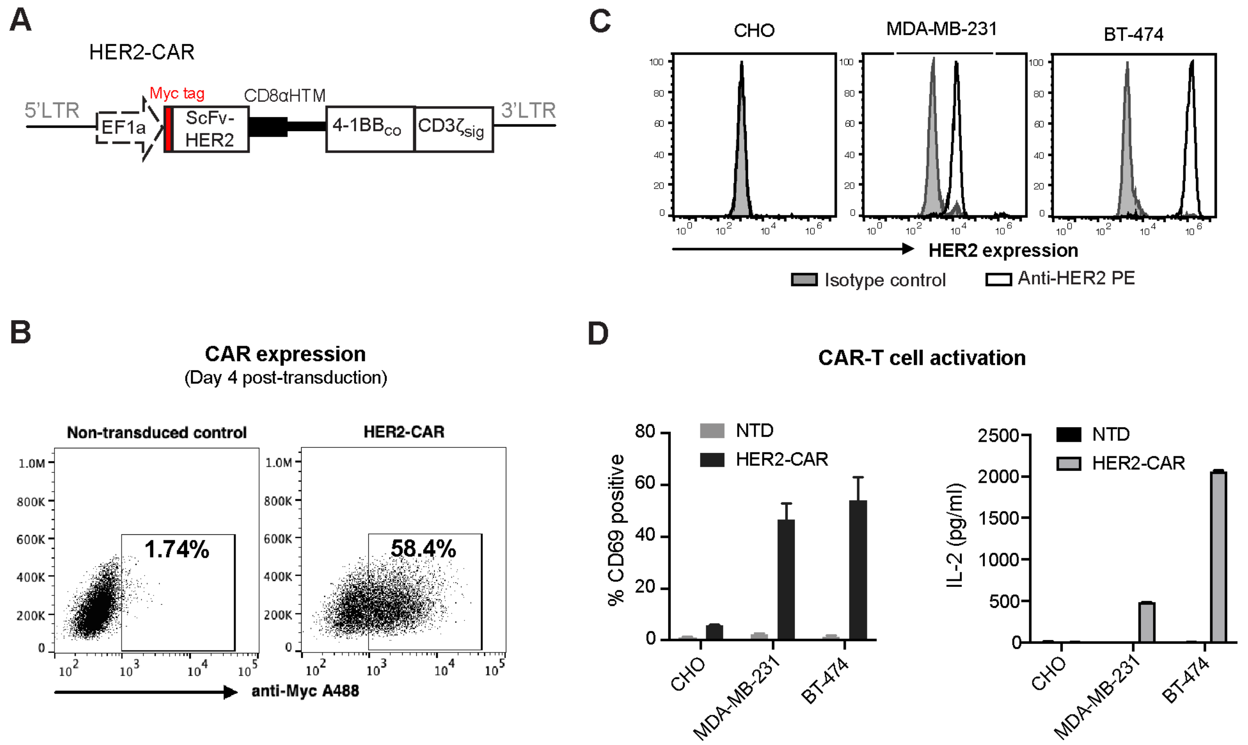

Figure S1. Validation of HER2-CAR Jurkat T cells. (A) Lentiviral vector encoding for HER2-CAR. **(B)** Expression and localization of CAR on the surface of Jurkat T cells at Day 4 post-transduction via flow cytometry. **(C)** Detection of HER2 surface expression on different target cell lines. Target cells were stained with Phycoerythrin (PE) conjugated α-HER2 antibody; light gray histograms denote unstained cells (control). **(D)** Non-transduced control or HER2-CAR T cells were co-cultured with different target cell lines at an Effector:Target (E:T) ratio of 3:1 for 24h. Activated T cells were stained for CD69 expression with Allophycocyanin (APC)-conjugated α-CD69 antibody and analyzed by flow cytometry. ELISA assay was also performed to quantify IL-2 cytokine secretion into the cell culture media. Data represents mean ± SD from 2 biological replicates.

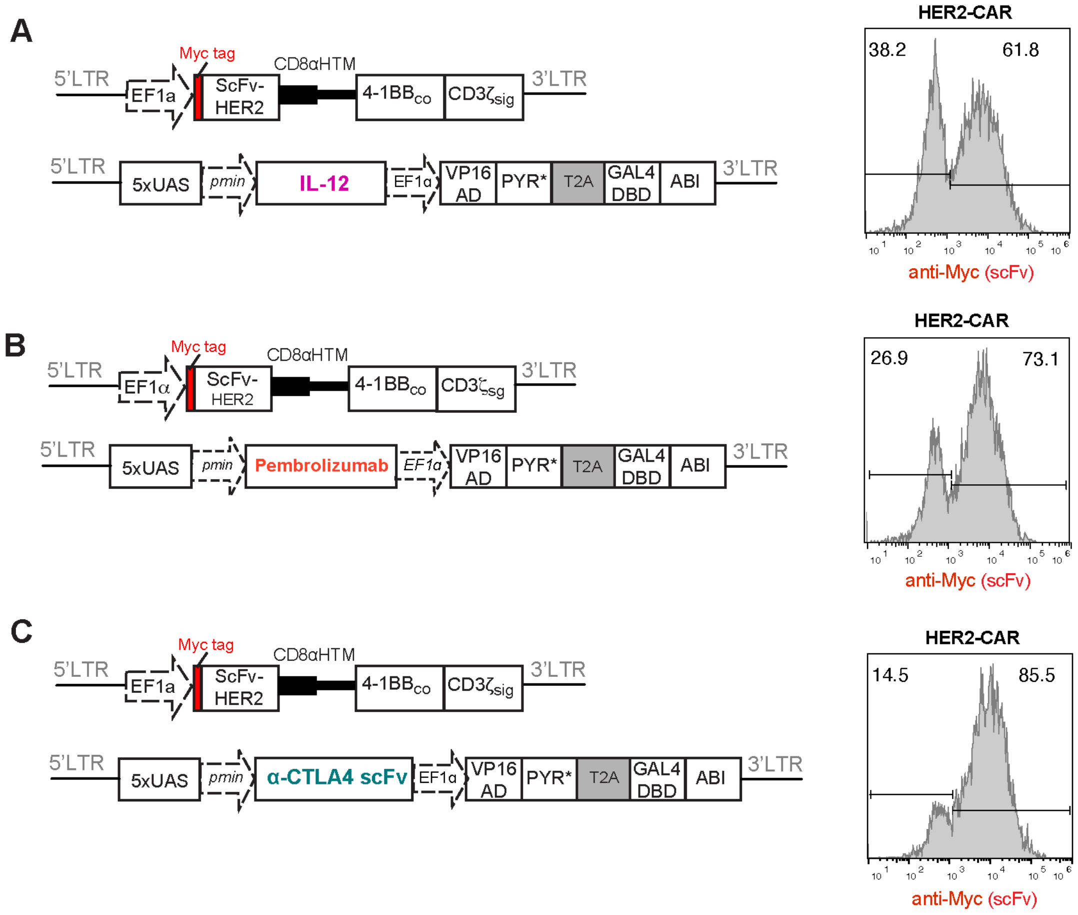

Figure S2. Surface expression of HER2-CAR in co-transduced Jurkat cells. Jurkat T cells were co-transduced to express HER2-CAR and the corresponding ABA-inducible therapeutic gene circuits including **(A)** IL-12, **(B)** Pembrolizumab, and **(C)** α-CTLA4 scFv. At day 4 post-transduction, HER2-CAR expression was detected by surface staining of transduced cells with anti-Myc tag antibody and analyzed by flow cytometry.

HRMS spectra (M+Na)

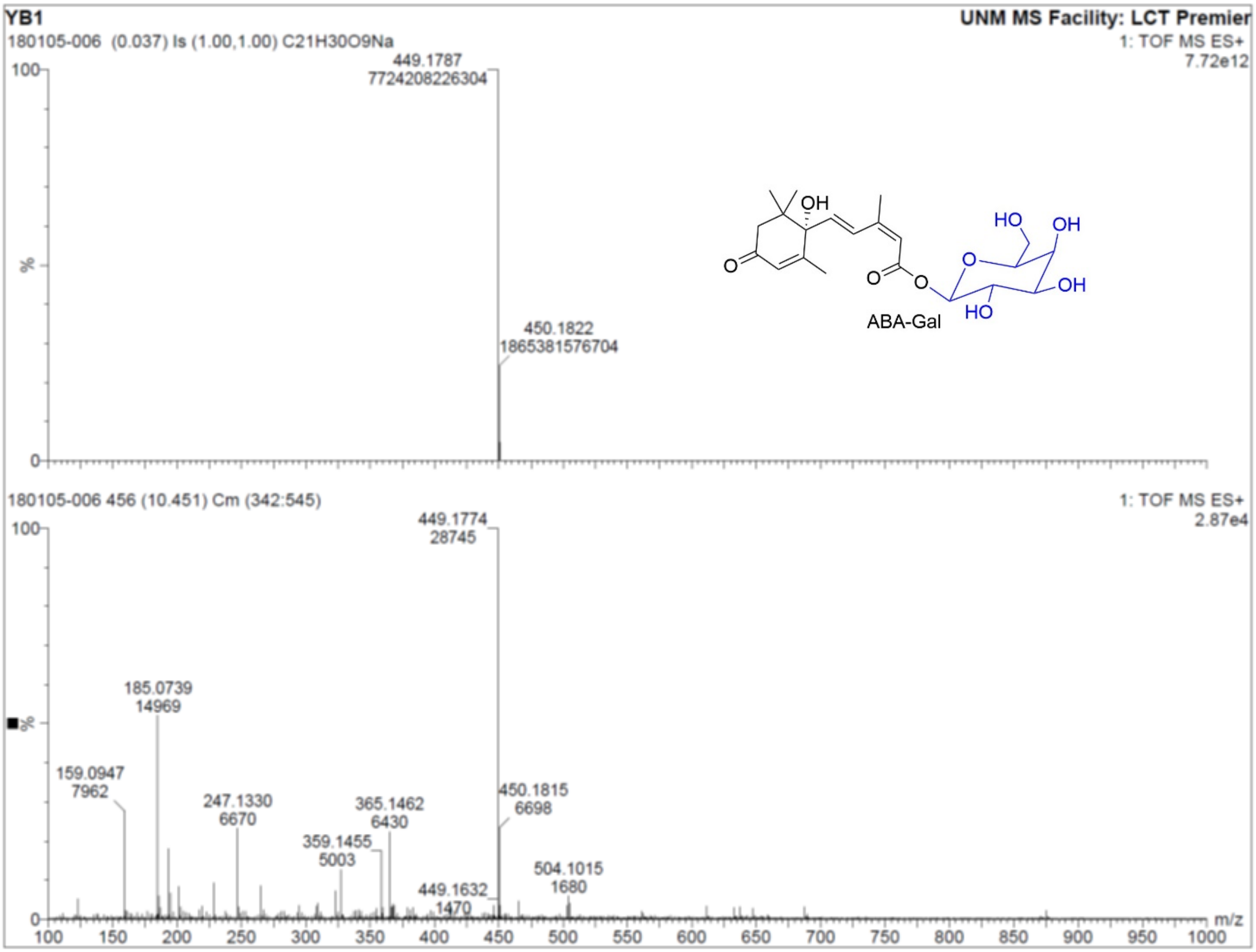

^{1}H and ^{13}C NMR spectra

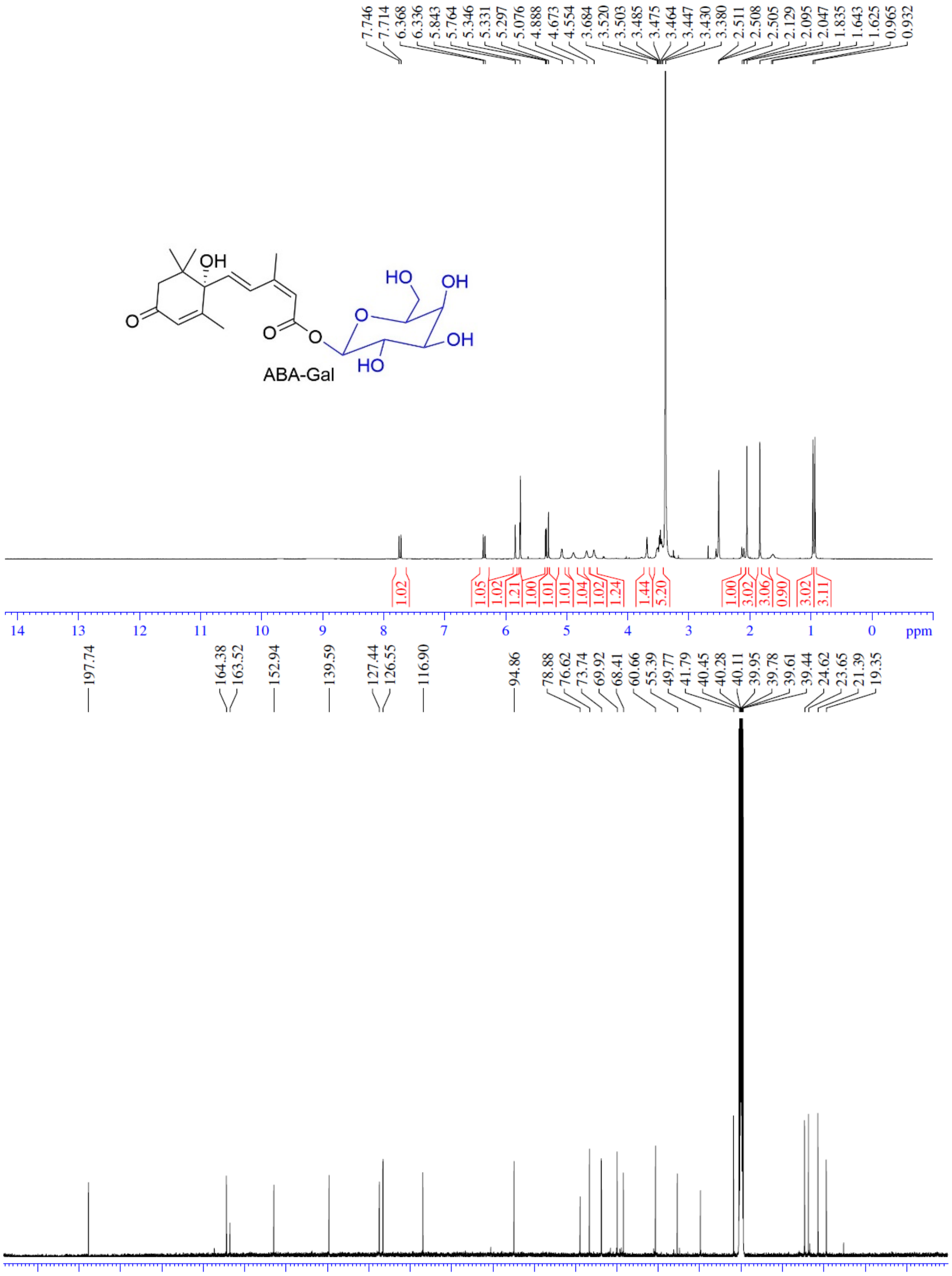

Table S1: Components of HER2-CAR and therapeutic protein plasmids

**Components**	**Sources**
CD8α signal sequence	MALPVTALLLPLALLLHAARP
Human IgK signal sequence	MDMRVLAQLLGLLLLCFPGARC
Myc tag	EQKLISEEDL
Flag tag	DYKDDDDK
α-HER2 scFv (clone 4D5-5)	Addgene plasmid # 85423
CD8α Hinge and Transmembrane domains	NP_001759.3 aa 138–206
41BB	UniProt - Q07011 aa 214-255
CD3ς	UniProt - P20963 aa 31-142
TRAIL	UniProt - P50591 aa 114-281
IL-12	Addgene plasmid # 85428
α-CTLA-4 scFv	Addgene plasmid # 85436
Pembrolizumab (α-PD1)	Addgene plasmid # 85436
